# A Participatory Comic Book Workshop to Improve Youth-Friendly Post-Rape Care in a Humanitarian Context in Uganda: A Case Study

**DOI:** 10.9745/GHSP-D-22-00088

**Published:** 2023-06-21

**Authors:** Carmen H. Logie, Moses Okumu, Miranda Loutet, Isha Berry, Alyssa McAlpine, Simon Odong Lukone, Nelson Kisubi, Simon Mwima, Peter Kyambadde

**Affiliations:** a Factor-Inwentash Faculty of Social Work, University of Toronto, Toronto, Canada.; b Centre for Gender & Sexual Health Equity, Vancouver, Canada.; c Women’s College Research Institute, Women’s College Hospital, Toronto, Canada.; d United Nations University Institute for Water, Environment, & Health, Hamilton, Canada.; e School of Social Work, University of Illinois at Urbana-Champaign, Champaign, IL, USA.; f Dalla Lana School of Public Health, University of Toronto, Toronto, Canada.; g Uganda Refugee and Disaster Management Council, Yumbe, Uganda.; h National AIDS Coordinating Program, Ugandan Ministry of Health, Kampala, Uganda.; i Most at Risk Population Initiative, Kampala, Uganda.

## Abstract

**Background:**

Graphic medicine formats, such as comic books in which health information is presented alongside images, may be a useful learning tool to improve post-rape care and youth-friendly service provision among health care providers in humanitarian contexts. We describe the development and pilot-testing of a workshop using a comic book to improve youth-friendly post-rape care with providers in Bidi Bidi refugee settlement, Uganda.

**Program Development and Piloting:**

We conducted 6 focus groups with refugee young men (n=3) and women (n=3) aged 16–24 years and 28 in-depth individual interviews (refugee youth: n=12; health care providers: n=8; elders: n=8). Findings informed the development of a workshop that included a participatory comic book on sexual and gender-based violence (SGBV) and youth, SGBV stigma, youth-friendly health care, and post-exposure prophylaxis. Comic book illustrations specifically addressed health care confidentiality and examples of being a supportive health care provider. Then, we conducted a 1-day workshop with health care providers (n=20) that included structured activities addressing SGBV impacts and related stigma and included comic book discussions. Open-ended survey data were collected 8 weeks after the workshop to explore health care providers’ experiences with the workshop, perceived impact of the intervention on their work, and support required to implement youth-friendly services for SGBV survivors. Qualitative data were analyzed using thematic approaches. Open-ended response data indicated that: comic book methods were informative and interactive; health care providers felt more empowered to offer youth-friendly services and spaces; and health care providers want additional SGBV training and institutional support for youth-friendly spaces and community engagement.

**Implications:**

A comic book intervention has the potential to meaningfully engage health care providers in humanitarian contexts to provide youth-friendly health care, acquire skills for engaging in SGBV prevention, create youth-friendly clinic spaces, and identify health care and community SGBV prevention needs.

## Abstract (precis)

Participatory comic books offer a novel approach to strengthening health care providers’ understanding of refugee youth’s post-rape care needs and can be embedded in provider training in humanitarian contexts.

## Teaser

**Key Findings:**

- Youth-friendly post-rape care services can increase engagement in health services among sexual violence survivors, yet there are knowledge gaps in effective training approaches in humanitarian contexts.
- Graphic medicine approaches, such as comic books, offer a medium for training health care providers to improve clinical care practices but are understudied in relation to post-rape care services in humanitarian contexts.
- Health care providers engaged in a participatory comic post-rape care intervention in a humanitarian context in Uganda reported that this approach facilitated the provision of youth-friendly health services and identified areas for future training and program development.

**Key Implications:**

- Qualitative data collection can help generate insights from multiple stakeholders on graphic medicine tools and inform the development and evaluation of youth-centered post-rape care training, particularly in humanitarian contexts.
- Innovative and low-cost methods for training, such as participatory comic books, can meaningfully engage health care providers in addressing the post-rape care needs among refugee youth in humanitarian contexts.

## BACKGROUND

The number of forcibly displaced persons—82.4 million—has never been higher.^1^ Yet, the sexual health needs of youth in humanitarian contexts remain understudied.^2^^,^^3^ This is also true regarding the readiness of health care providers (HCPs) to offer youth-friendly post-rape clinical care services in humanitarian contexts. Post-rape clinical care includes access to post-exposure prophylaxis (PEP), testing for HIV and other sexually transmitted infections, emergency contraception, and linkages to psychosocial support and legal services. Hosting more than 1.4 million refugees, Uganda is an important context to understand HCPs’ post-rape care service provision for refugee youth. However, there is limited research on effective training approaches for youth-friendly post-rape care provision in humanitarian contexts.

Provision of survivor-centered post-rape care is a priority area in the Minimum Initial Service Package for sexual and reproductive health (SRH), which outlines key areas for service provision within humanitarian contexts.^4^ The Minimum Initial Service Package recommends sexual and gender-based violence (SGBV) prevention and survivor-friendly clinical care, including a focus on confidentiality and a code of conduct for HCPs.^4^ HCPs should be trained to provide psychosocial support strategies (e.g., empathy), survivor-centered practices that respect rights and agency, and clinical service access (e.g., PEP).^4^ In addition, SRH services need to be responsive to the needs of youth in humanitarian contexts facing unique economic (e.g., unemployment), relational (e.g., loss of family), and sociocultural (e.g., forced marriage) vulnerabilities that elevate SGBV exposure while reducing access to post-rape care.^4^^,^^5^ Thus, it is recommended that youth participate in the design and implementation of services to ensure that post-rape care addresses their needs.^4^ Barriers to the implementation of youth-tailored post-rape care in humanitarian contexts include cultural norms (e.g., stigma) and financial and other structural constraints.^3^^,^^6^ A systematic review by Singh et al. notes the urgent need for research on SGBV prevention and post-rape clinical care in humanitarian contexts,^3^ particularly with youth.^2^^,^^3^

Health care providers should be trained on providing survivor-centered post-rape care that respects right and agency and is responsive to the needs of youth in humanitarian contexts.

Training on youth-friendly approaches is needed among HCPs providing post-rape care within humanitarian contexts.^7^ Such youth-friendly approaches include service plans that address youth health needs, institutional support for youth-friendly services, and youth and community engagement in service planning and provision.^7^ Recent systematic reviews on youth-friendly SRH service access and uptake spanning global regions identified the need for intensive HCP training.^8^^,^^9^ Further information on effective strategies to train HCPs in humanitarian contexts is required to ensure youth-friendly post-rape care experiences.^9^

The need for youth-friendly post-rape care training with HCPs has been identified in East African contexts,^10^^,^^11^ including within conflict-affected regions.^3^^,^^12^^,^^13^ A study with HCPs from conflict-affected Gulu District in Uganda found that inadequate training opportunities and policies within health facilities contributed to a lack of confidence when working with SGBV survivors.^12^ HCP training needs for working with SGBV survivors have also been identified in Kenya^14^ and Tanzania.^11^ In Kenyan humanitarian^13^ and non-humanitarian^10^ contexts, SGBV survivors reported concerns regarding HCP confidentiality and fears of community stigma.^13^ A recent study with women sexual violence survivors from Somalia and Syria noted the need for additional research to understand sexual violence stigma in health care.^15^

Graphic medicine, which presents health information alongside images and text, is a possible approach to enhance HCP training to improve post-rape care and youth-friendly service provision among HCPs in humanitarian contexts.^16^ Graphic medicine formats, such as comic books, that visually depict emotional experiences and concepts may be a useful learning tool to help HCPs understand the depth of patient experiences.^16^^,^^17^ Comic books have been used within medical education to build empathy, communication skills, understanding, and patient-centered care in Canada^18^^,^^19^ and in the United States.^20^ A study with HCPs in South Africa found that animated storybooks helped HCPs acquire confidence and skills to provide child-friendly HIV services.^21^ Together, this evidence signals the utility of comic books in HCP training, but further attention to sub-Saharan African contexts is needed.

Little is known about the use of graphic medicine as a tool to improve post-rape care and youth-friendly services with HCPs in humanitarian contexts. To illustrate the utilization of graphic medicine approaches within these settings, we describe the development and pilot testing of the *Ngutulu Kagwero* (“agents of change” in Bari) intervention to reduce sexual violence stigma, increase bystander practices to prevent SGBV, and increase the provision of youth-friendly post-rape clinical services among HCPs in Bidi Bidi refugee settlement, Uganda.

## PHASE 1: NGUTULU KAGWERO INTERVENTION DEVELOPMENT PROCESS

To develop the intervention, we engaged HCPs working in Bidi Bidi refugee settlement, Uganda, in a 2-phase sequential transformative qualitative pilot study of Ngutulu Kagwero.^22^ Phase 1 and Phase 2 activities were conducted in collaboration with the Uganda Refugee and Disaster Management Council, a community-based agency focused on refugee youth service provision in Bidi Bidi. Both phases involved working with a team of 8 peer navigators (4 young women and 4 young men), refugee youth aged 16–24 years living in Bidi Bidi who provided feedback about the study design, helped with the interpretation of findings, and contributed to comic book design, intervention development, and supported workshop implementation.

### Interviews and Focus Group Discussions

During February 2020, Phase 1a involved a formative qualitative phase with 6 focus groups that included 48 refugee young men (n=3) and young women (n=3), and 28 in-depth individual interviews (IDIs; 12 with refugee youth sexual violence survivors, 8 with HCPs, and 8 with elders). There was equal gender distribution within focus groups and the youth IDIs. Participants received an honorarium equivalent to CAD$13 (38000 Ugandan shillings [Ush]). Participant inclusion criteria were being able to provide their own informed consent and speak and understand a study language (i.e., Juba Arabic, Bari, or English). Inclusion criteria for youth were aged 16–24 years, identified as a refugee, and lived in Bidi Bidi. Congruent with Ugandan laws and aligned with research on the importance of youth agency and participation in sexual health research and potential barriers requiring parental consent,^23^^–^^25^ youth aged 16–17 years provided their own research consent without parent approval. Elder inclusion criteria were aged 55 years or older, identified as an elder in their community, and lived in Bidi Bidi. For HCPs, inclusion criteria were aged 18 years or older and able to provide health care services (e.g., nurse, clinical officer, or midwife) in Bidi Bidi.

Focus group discussions (FGDs) and IDIs followed a semistructured interview guide (Supplement) that explored experiences of sexual violence and post-rape health care-seeking practices and experiences among refugee youth in Bidi Bidi. For example, youth and elders were asked, “What health services exist to help address these problems of violence?” To facilitate participant engagement in problem-solving, interviewers asked: “How can these efforts be improved?” with specific prompts about respondents’ hopes for HCP interactions with SGBV survivors. Within the HCP interviews, participants were asked about the process of post-rape care services at their place of work (e.g., “What is the process like for receiving post-rape care in your clinic/setting?”). HCPs were also asked to reflect on barriers and facilitators for youth to access post-rape care services and their ideas for improvements. Questions included: “What are some barriers to survivors accessing this care?”

The mean age of HCP IDI participants (n=8) was 30.1 years (standard deviation [SD]: 3.8 years). HCP participants included women (n=5) and men (n=3), and most (n=5) had more than 3 years of work experience, with the remaining (n=3) employed 1–3 years. Participant roles included midwives (n=4), clinical officers (n=2), laboratory technologists (n=1), and nurses (n=1).

Among youth IDI and FGD participants (n=60), the mean age was 20.7 years (SD: 2.16 years), half were young women (n=30) and half young men (n=30), and most were from South Sudan (n=50) with the remaining from the Democratic Republic of Congo (n=10). Eight elders participated in IDIs (mean age 58.2 years, SD: 3.88 years); half were women (n=4), half were men (n=4), and most were from South Sudan (n=7), with 1 from the Democratic Republic of Congo.

### Qualitative Findings

From March to June 2020, FGD and IDI transcripts were audio-recorded, transcribed verbatim, translated into English, and reviewed by the Ugandan study team to ensure the meaning was correctly conveyed in the translation process. Transcripts were analyzed by 3 team members using thematic analysis, a method involving multiple reviews of transcript text, assigning codes, creating themes and subthemes, and finally refining and organizing themes into an overarching narrative.^26^ All analyses were conducted using Dedoose version 8.0.35 (SocioCultural Research Consultants). Member checking included our team of peer navigators to ensure the themes were adequately captured.

Participant narratives revealed that post-rape health care needs and priorities among refugee youth included: improved confidentiality; reduced stigma in health care encounters; enhanced access to SRH services; and mental health support. The identified themes and qualitative content were used to inform the HCP comic books, workshop, and intervention.

#### Improved Confidentiality

**Figure:**
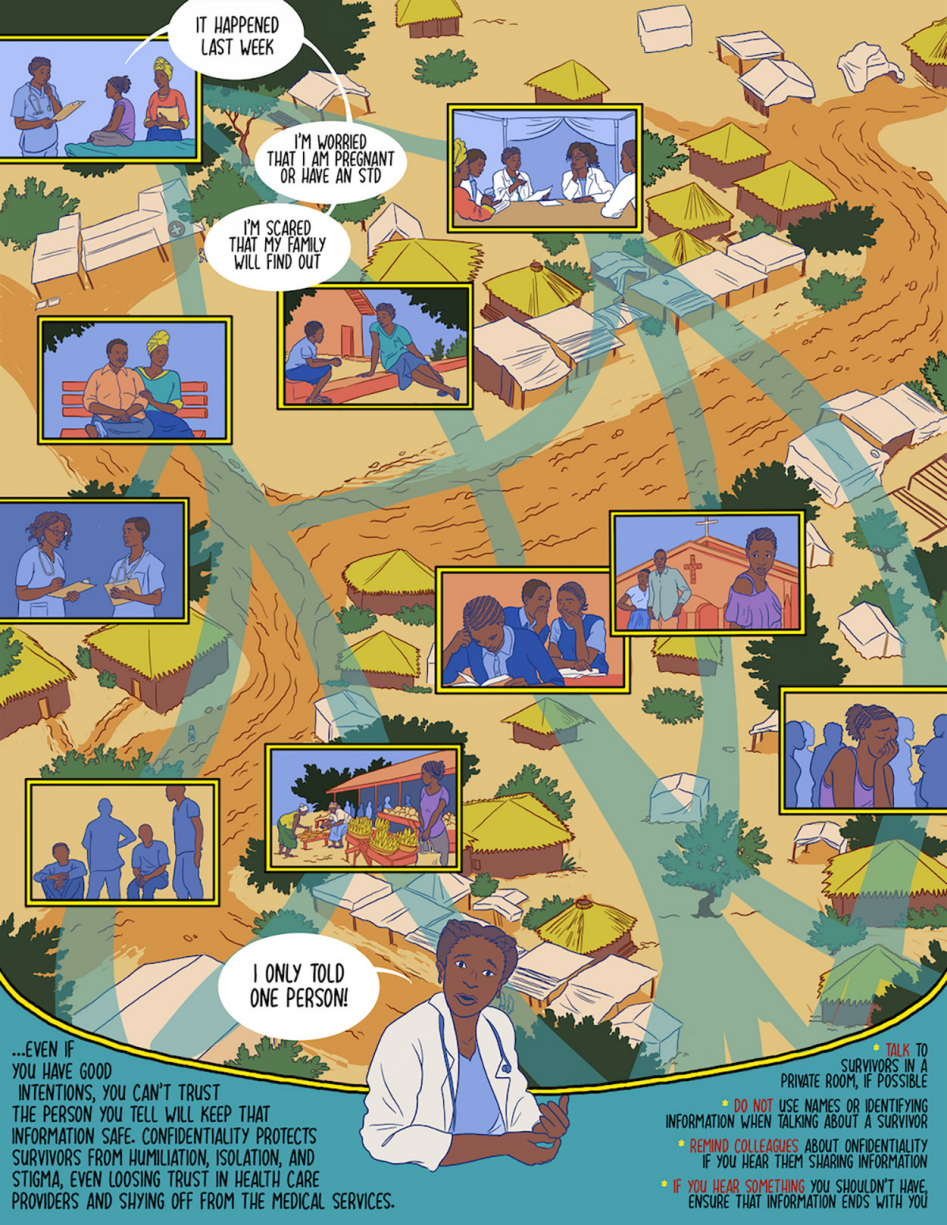
Comic book example on health care provider confidentiality for the workshop.

Youth narratives illustrated both the challenges with and importance of confidentiality in health care encounters. A young man described confidentiality breaches with harmful repercussions.

*There is no confidentiality among the community members and service providers. They get infected with [sexually transmitted infections], HIV, and other diseases. The community criticizes a lot, calling the survivor names because they believe the girls are always trying to seduce the boys.* —Youth, man, FGD

Youth narratives illustrated both the challenges with and importance of confidentiality in health care encounters.

This narrative revealed how HCP confidentiality breaches could lead to community-wide stigma, particularly toward women and girl survivors. These confidentiality concerns and fear of community reactions were corroborated by a young man.

*They say going to the health center will just expose them to a scorning community… So, they rather keep quiet and everything disappears in thin air. At least no one will ever know she was raped.* —Youth survivor, man, IDI

This narrative also suggests that fear of judgment will prevent sexual violence disclosure and subsequent care access. An elder corroborated this narrative.

*Some [survivors] failed to get accurate counseling to help them recover because some health workers are not trained to give counseling and even lack [adherence to] confidentiality code of conduct.* —Elder, woman, IDI

Conversely, HCP confidentiality can enhance trust and sexual violence disclosure.

*They [HCPs] need to keep secret and confidential information of the survivors so as to build trust in them.* —Youth, woman, FGD

HCP narratives aligned with these perspectives on the importance of improved confidentiality in reducing barriers to post-rape care.

*This is one of the things that might make the survivor more likely to look for or use services because they know that their information will not spread to everyone.* — HCP, woman, IDI

#### Reduced Stigma in Health Care Encounters

Participants described existing stigma during interactions between youth and HCPs that may present barriers to seeking post-rape care. HCPs mentioned the need to educate providers on not blaming survivors or treating them harshly so that survivors do not fear them. Another HCP suggested that these negative attitudes may make youth feel stigmatized and isolated for seeking violence-related services. The need for changes in health care settings was also raised by an HCP woman who discussed the importance of creating youth-friendly environments so that survivors will feel welcome to talk.

To improve HCP attitudes and reduce stigma, participants described enhancing practice skills and training needs. For instance, an HCP suggested that providers need to listen well, maintain client confidentiality, and provide health education.

A young woman survivor suggested the need for stigma education for HCPs, specifically on how to support survivors going through the psychological trauma they experience when they feel rejected by their community.

#### Enhanced Access to SRH Services

Narratives highlighted the need to address several barriers to accessing SRH services, including sexual violence reporting requirements, transportation, and logistical barriers. First, youth participants described being asked by HCPs to prove they were raped.

*The health workers are normally willing to listen and give services, but at times, they want you to prove that you were raped. How do you prove?* —Youth survivor, woman, IDI

Narratives highlighted the need to address several barriers to accessing SRH services, including sexual violence reporting requirements, transportation, and logistical barriers.

Another youth spoke of delays in receiving SRH services due to the lengthy reporting process.

*When I get raped, I need emergency pills and even the one which prevents HIV and AIDS—yes, I need PEP to prevent getting HIV. But whenever you go to report, they take long asking many questions and recording.* —Youth survivor, man, IDI

Another commonly reported barrier was transportation to clinics.

*Provide accessibility to services to health services. People should not move very far distances to seek medical care. For instance, currently a women raped in village 11 will move through 6 villages to reach village 5 where there is a health facility. This distance is too long and difficult to walk*. —HCP, woman, IDI

Youth also described that closer proximity to health facilities from one’s residence would make it easier for survivors to access SRH services.

*[Make] health workers and community leaders readily available to victims, maybe by availing toll-free numbers and consultation desks where they can easily access help*. —Youth survivor, man, IDI

Participants also discussed insufficient resources. An HCP noted the need for having adequate drugs and testing kits and integrating SGBV services in a central location for easier access. A young man reported the need for more health centers and police help desks to help with emergencies and transportation difficulties. Another participant highlighted the need for increased youth-friendly services and community education to raise community awareness about the importance of seeking health care services when they experience SGBV. Others noted language barriers to accessing services because most victims do not speak English.

#### Mental Health Support

Participants discussed refugee youths’ unmet mental health needs and the importance of SGBV counseling.

*I have been going for counseling in the health center, and they were really nice to me. Sometimes we share experiences with others which helps us.* —Youth survivor, woman, IDI

Other youth underscored the importance of HCPs engaging in training for counseling youth SGBV survivors.

*There should be a contact person with a team that should be trained to give psychosocial support to the survivors because they need it to recover from the horrific incidence.* —Youth, man, FGD

Others discussed the need for ongoing mental health support after reporting sexual violence.

*Messages about SGBV can be included in these teachings to health workers and conveyed to the recipients. Health workers need to appreciate the post-rape stress and how to deal with it. They should have counseling sessions in follow-up visits, not only to see the victims once on the day they are examined in the presence of police.* —Youth survivor, man, IDI

This need for additional mental health support was confirmed by an elder who stated that survivors only get counseled when they go to the hospital.

In addition, HCP capacity is a challenge within the community.

*Sometimes the service providers are never available in time to listen or counsel victims.* —Youth survivor, man, IDI

This mental health support could extend beyond the clinic. One HCP noted the need to train more counselors and post them in villages. Another youth participant suggested helping victims easily access help from health workers and community leaders by using toll-free numbers and consultation desks.

### HCP Comic Book and Intervention Development

Taking these findings, the team worked with an artist and peer navigators to develop a comic book for the workshop. Two scenarios in the comic book explicitly addressed HCP practices for post-rape care. The first comic book was developed based on the qualitative theme of improving confidentiality. HCPs, youth, and elders agreed that the lack of confidentiality within health care settings was a significant barrier among survivors when seeking post-rape care. Through the creation of this comic book, the content encourages HCPs to uphold survivors’ confidentiality due to the negative impacts that privacy breaches have on survivors and within the community. One HCP described the need to assure survivor confidentiality to encourage them to visit the health center for treatment and other issues. The comic book shows the importance of confidentiality in supporting youth survivors through the illustrated negative experience of the protagonist when an HCP disclosed her health care information to others.

The comic book content encourages HCPs to uphold survivors’ confidentiality.

The second scenario was inspired by the qualitative themes of enhanced access to SRH services and mental health support. Participants described barriers to post-rape care that included unsupportive approaches of HCPs and challenges when seeking SRH services. Addressing these barriers can improve post-rape care experiences.

**Figure:**
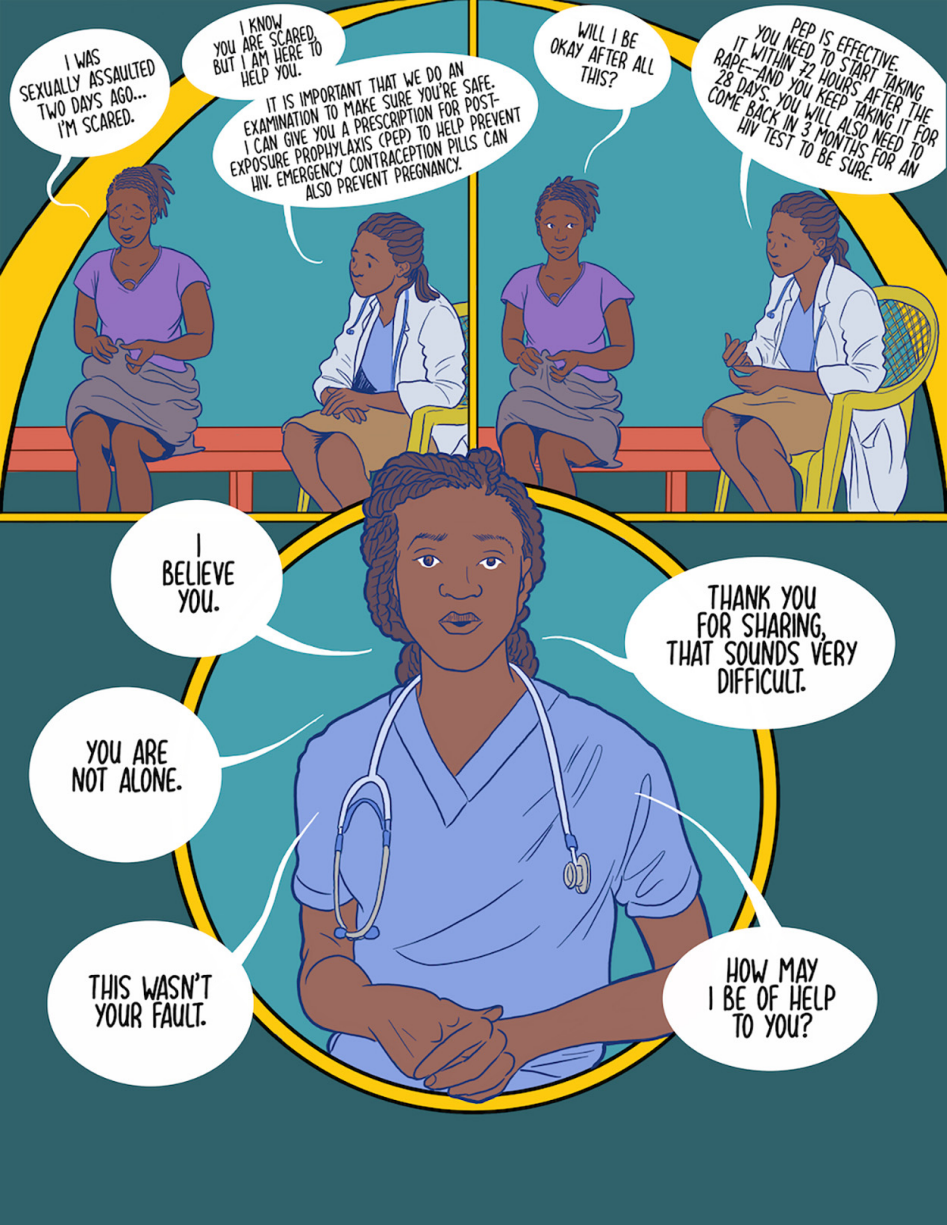
Comic book example on health care provider’s youth friendly post-rape care for the workshop.

*[I need] good or proper counseling training so that I provide counseling better to these persons so that they will not lose hope in them and make them believe that the world still needs them. They should not feel isolated or rejected from the community*. —HCP, woman, IDI

The comic book provides examples of both supportive and nonsupportive responses to youth SGBV violence survivors and provides suggestions for how to provide supportive post-rape care through affirming statements and SRH education.

Details on the workshop development, content, and structure have been described elsewhere.^22^ In brief, the workshop was a single-day 6-hour session with structured activities on group norms, reducing stigma toward SGBV survivors,^27^ SGBV and its impacts,^28^ PEP knowledge and awareness,^28^^,^^29^ bystander intervention and role-plays,^30^ and comic book reading and discussion. The full comic book, developed from Phase 1 qualitative data, addressed peer support, peer gossip, HCP confidentiality, being a supportive HCP, being a supportive police officer, sexual violence stigma, positive masculinities, and family support.^22^ Participants received 2 comic book versions: one with dialogue and one blank to create their own dialogue.

## PHASE 2: INTERVENTION PILOT TESTING

### Participants

Phase 2 involved pilot testing the workshop at a community center in Bidi Bidi from November 2020 to December 2020. HCPs (n=20) participated in a tailored 1-day workshop on the impact of SGBV on refugee youth, provision of youth-friendly post-rape care, PEP knowledge and awareness, and sexual violence stigma. Inclusion criteria for HCP workshop participants included being aged 18 years or older, providing health care services in Bidi Bidi and/or to refugees in Yumbe, capable of providing informed consent, and being able to speak and understand a study language. We used peer-driven purposive and convenience sampling to recruit HCPs. Each participant was given an honorarium of CAD$35 (USh96000) (CAD$20 for workshop, CAD$15 for surveys) and food and refreshments during the workshop. All consent forms and study materials were translated into the study languages, and all participants gave their written informed consent.

### Data Collection

At baseline, tablet-based surveys were conducted to collect sociodemographic data, including gender, age, type of health care facility and role, as well as experience with providing health care services to youth, refugees, and/or those who experienced SGBV. Open-ended responses were collected 8 weeks after workshop completion to 3 questions that focused on (1) workshop experiences: What was your experience with the sexual and gender-based violence workshop that you participated in 2 months ago?; (2) perceived impact of the intervention on their work: What changes have you been able to implement following the workshop you participated in?; and (3) support required to implement youth-friendly services for SGBV survivors: What support might you need to implement further youth-friendly services?

All HCP participants reported fluency and comfort in answering these questions in English, and responses were audio-recorded on the tablet using the SurveyCTO software and transcribed verbatim.

### Ethical Approval

The study was approved by the Mildmay Uganda Research Ethics Committee (REF 0212-2019), Uganda National Council for Science and Technology (SS 5273), and the University of Toronto (37981).

### Workshop Findings

All HCP (n=20; 100%) participants completed the sociodemographic measures (Table). All participants had experience providing services to refugees aged 16–24 years, and 60% (n=12) had experience providing services to youth SGBV survivors (Table).

**TABLE.** Sociodemographic Factors of Health Care Providers Enrolled in Phase 2 of Ngutulu Kagwero Comic Book Workshop, Uganda

	**No. (%)**
Total	20
Age at baseline, years, mean (standard deviation)	30.3 (4.2)
Gender	
Woman	8 (40.0)
Man	12 (60.0)
Role	
Clinical officers	6 (30.0)
Nurses	5 (25.0)
Lab technician/assistant	5 (25.0)
Midwife/community health worker	5 (25.0)
Experience, years	
1–3	10 (50.0)
More than 3	10 (50.0)
Provide services to refugees aged 16–24 years	
Very often/often	20 (100.0)
Not often/never	0 (0)
Provide services to adults having experienced sexual and/or gender-based violence	
Very often/often	7 (35.0)
Not often/never	13 (65.0)
Confidence in providing services to adults having experienced sexual and/or gender-based violence	
Very confident	12 (60.0)
Confident	8 (40.0)
Not confident/not at all confident	0 (0)
Provide services to youth having experienced sexual and/or gender-based violence	
Very often/often	12 (60.0)
Not often/never	8 (40.0)
Confidence in providing services to youth having experienced sexual and/or gender-based violence	
Very confident	13 (65.0)
Confident	7 (35.0)
Not confident/not at all confident	0 (0)

Key themes from HCP responses (n=20) to the open-ended questions post-intervention included knowledge gained through the training on sexual violence and supporting survivors; initiatives following workshops, including youth-centered strategies to promote privacy; and additional HCP training support needs and resources for providing post-rape care and engaging in SGBV prevention with refugee youth (The Supplement contains HCP responses on these themes).

#### Workshop Learning Outcomes

Participants shared how the comic book format was helpful in increasing their knowledge about sexual violence in general. Others discussed acquiring knowledge of appropriate responses to instances of SGBV or sexual harassment, including how to identify, report, and assist and support survivors. Participants indicated how this knowledge helped them to better support survivors.

*I learned a lot in SGBV management and how to help a client in case they need my help.* —HCP, woman

Others specifically noted learning more about linkage to post-rape clinical care, including providing support, HIV testing and PEP, and other medicine. Further, participants described how the workshop increased sexual violence prevention knowledge and helped them understand their responsibilities as bystanders. Participation in the workshop also helped HCPs gain knowledge, strengthen clinical skills, and build confidence in the management of SGBV cases.

Participation in the workshop also helped HCPs gain knowledge, strengthen clinical skills, and build confidence in the management of SGBV cases.

Regarding the use of the comic books within the workshop, participants described this methodology as “educative and interactive” and that “the comic book was so interesting and it helps to ease a teaching session.”

Participants reported that comic books, in conjunction with the workshops, were an effective medium for delivering information about SGBV.

*It provided a platform for experience sharing and the comics was a new tool that is going to be effective communication of sexual and gender-based violence messages. —*HCP, man

In the future, other versions of graphic medicine may be considered for participants with lower levels of literacy. For example, an HCP suggested that videos would be helpful for those who cannot read.

#### Initiatives Following Workshops

Participants described various youth-friendly initiatives, such as youth clinic days, that they created following workshop participation. Others tried to reduce barriers for youth accessing care through efforts to ensure confidentiality. A participant described strategies to foster increased professional competency and privacy within the workplace.

*My staff were able to allocate a private corner for the management of adolescents and sexual assault survivors. And also, we are able to now conduct training, build confidence among the staff and me too, so that we are able to manage cases in a confidential manner and provide appropriate information to survivors.* —HCP, woman

Participants noted a renewed focus on SGBV education at health clinics. Another HCP described how new services were initiated to respond to youth’s mental health needs.

*We have been able to start counseling sessions for youth at the youth-friendly corner. HCPs specifically attend to the youth database. It’s currently very active. It is doing well.* —HCP, woman

#### Further Support Needed for Youth-Friendly Services

Participants reported needing additional resources to optimize youth-friendly care in their clinical contexts. These included resources needed to provide information and comfortable spaces in clinics.

*There is need for IEC [information, education, communication] materials, such as posters, signposts that can communicate on sexual gender-based violence. There is need for TV with entertainment. We need a separate laboratory for youth. And we need more comic books that teach us about SGBV.* —HCP, woman

Others indicated that HCPs would benefit from more education, such as SGBV trainings, so that they can better assist youth.

Some HCPs discussed limited finances as a structural limitation to implementing youth-friendly services in their clinics and the need for financial support. Further, HCPs noted the need for recreational activities to help with youth education and service engagement.

Participants also discussed the importance of engaging the broader community in supporting SGBV prevention and survivors.

*There is a need for strengthening community awareness, as this can constantly help us, help the community to identify issues of gender-based violence in the communities, and probably talk to the community leaders also, as they are the main, they are the pillars of the community who can help identify these cases [of SGBV] and bring them out.* —HCP, man

HCPs also shared ideas for future directions for youth-friendly SRH care. These include SRH self-care strategies, including HIV self-testing to reduce youth fear and stigma related to disclosing that they want to get tested. Others also expressed the need for more SRH training to facilitate youth-friendly service provision.

## LESSONS LEARNED

- Graphic medicine formats, such as participatory comic books, can be an effective tool to increase HCPs’ knowledge of youth-friendly post-rape care, including how to respond to and support refugee youth SGBV survivors.
- Graphic medicine was an engaging and informative tool for HCPs within the context of a humanitarian setting in Uganda. While comic books can be modified to all levels of literacy, other graphic medicine formats, such as videos, can also be considered.
- Experiences of HCPs within our intervention indicate that comic books can be a feasible method of youth-friendly post-rape care training. Engaging HCPs in post-rape care knowledge-building interventions, such as our Ngutulu Kagwero workshop, may increase knowledge and skill competencies when working with young SGBV survivors in humanitarian contexts.
- Workshops aimed at improving post-rape care can integrate participatory, community-driven, and youth-friendly strategies within health care settings. HCPs participating in Ngutulu Kagwero reported improved confidentiality procedures and youth-centered programming within their settings.

### Limitations

The workshop was a nonrandomized, single-group pilot study with a small sample size not powered to detect changes in outcomes. Hence, future randomized controlled trials with larger sample sizes are warranted to assess intervention effectiveness. Longitudinal studies can assess if changes are sustained, and findings can be linked with clinic-level data to understand if HCP changes are associated with increased refugee youth clinic engagement, service uptake, and satisfaction. In addition, as our findings were retrieved from open-ended survey questions with HCPs, future research may benefit from observational data on service provision within health settings to identify youth experiences and further HCP training needs related to youth-friendly post-rape care. Future research could also conduct in-depth longitudinal interviews with HCPs to elicit richer feedback. The multiphase approach provided opportunities for collecting qualitative data from several stakeholders, generating arts-based products (comics, as shown), and collecting survey data with open-ended responses.

## SUMMARY AND CONCLUSION

Post-rape health care needs and priorities among refugee youth in this study include confidentiality, reduced stigma, and increased access to SRH and mental health services. To address these challenges, we developed and pilot-tested a participatory comic book intervention for HCP to increase youth-friendly post-rape care. Qualitative feedback revealed that the comic book and workshop helped HCP to feel more equipped to serve youth SGBV survivors. Yet HCP identified further training and institutional support needs to offer youth-friendly spaces and community engagement. Together, the study findings signal the promise of comic book interventions as tools for building HCP capacity for youth-friendly post-rape care with refugee youth in Bidi Bidi.

The study findings signal the promise of comic book interventions as tools for building HCP capacity for youth-friendly post-rape care with refugee youth in Bidi Bidi.

These findings align with calls for adolescent and youth-focused,^2^^,^^3^ survivor-centered post-rape care^4^ in humanitarian contexts. Similar to prior research in humanitarian contexts, our findings highlight that confidentiality^13^ was a primary concern among youth accessing post-rape care services, interlinked with stigma toward sexual violence survivors.^31^ Participants noted distance and transport costs as barriers to accessing post-rape care, corroborating prior research on economic vulnerabilities that hinder refugee youth SRH access.^4^^,^^5^ Our findings suggest that HCP participants acquired knowledge and skills regarding survivors’ rights and SGBV clinical services, as recommended in the Minimum Initial Service Package,^4^ alongside awareness and strategies to address confidentiality concerns. Additionally, HCP participants discussed acquiring more confidence following the training, which is notable as prior work in Northern Uganda noted a lack of confidence in post-rape care service delivery due to unmet HCP training needs.^12^

The open-ended responses after the workshop revealed an increased understanding of youth-friendly service provision alongside actions taken to improve SGBV and post-rape care services among HCP workshop participants. This feedback revealed increased attention to addressing youth-friendly spaces, stigma, and SGBV screening. Also aligned with the literature, participants identified the need for additional community engagement and institutional support.^7^ In particular, participants noted the need for financial support to implement, sustain, and grow youth-friendly services: financial constraints are a chronic challenge for youth-tailored SRH services in humanitarian contexts.^3^^,^^6^

Applying a participatory approach to graphic medicine using a comic book to address post-rape care and SGBV prevention with HCPs in humanitarian contexts is novel and holds promise in improving youth-friendly care, coping, and SGBV prevention. As shown in different contexts with other health issues, this graphic medicine format can foster greater HCP empathy, person-centered care, skills, and confidence.^16^^–^^19^^,^^21^ As discussed by participants and echoing the larger literature, additional funds and resources are needed beyond the 1-day workshop for information and skills spanning familial, community, and structural environments in humanitarian settings.^3^^,^^6^ Future research can address self-care strategies, such as HIV self-testing, noted by HCPs and understudied in humanitarian contexts.^32^ Innovative and low-cost methods, such as participatory comic books, can meaningfully engage both HCPs in addressing the SRH and rights of youth in humanitarian contexts.

## Supplementary Material

GHSP-D-22-00088-supplement.pdf

## Notes

**Peer Reviewed**

**First published online:** May 11, 2023.
