# Supplementary material for: A Participatory Comic Book Workshop to Improve Youth-Friendly Post-Rape Care in a Humanitarian Context in Uganda: A Case Study
Source: Glob Health Sci Pract. 2023 Jun 21;11(3):e2200088. doi: 10.9745/GHSP-D-22-00088 (PMC10285726; doi:10.9745/GHSP-D-22-00088)
Supplement: GHSP-D-22-00088-supplement.pdf [file GHSP-D-22-00088-supplement.pdf]

Supplement to: Logie CH, Okumu M, Loutet M, et al. A participatory comic book workshop to improve youth-friendly post-rape care in a humanitarian context in Uganda: a case study. *Glob Health Sci Pract*. 2023;11(3). https://doi.org/10.9745/GHSP-D-22-00088

Supplement. Focus Group and Interview Guides

Focus Group Guide

1. What is the situation of sexual and gender-based violence like in Bidi Bidi settlement? Can you give examples of what sexual and gender-based violence might look like in your community?
2. What are community attitudes toward sexual and gender-based violence?
3. What are some of the causes behind sexual and gender-based violence in your community?
4. Who is most vulnerable to sexual and gender-based violence in your community?
5. What are some challenges that survivors of sexual and gender-based violence face?
    a. PROBE: physical, psychological, social
6. What are some coping strategies survivors of sexual and gender-based violence use?
7. What are community responses when sexual and gender-based violence occurs?
    a. What is done to help survivors?
8. What is done to prevent violence in your community?
    a. How could these efforts be improved?
9. What social and legal services exist to help address these problems? Who provides these services?
10. How could these efforts be improved?
11. What would you like to see happen to reduce sexual and gender-based violence in your community?

Supplement to: Logie CH, Okumu M, Loutet M, et al. A participatory comic book workshop to improve youth-friendly post-rape care in a humanitarian context in Uganda: a case study. *Glob Health Sci Pract*. 2023;11(3). https://doi.org/10.9745/GHSP-D-22-00088

Interview Guide - Elders

1. What is the situation of sexual and gender-based violence like in Bidi Bidi settlement? Can you give examples of what sexual and gender-based violence might look like in your community?
2. What are community attitudes toward sexual and gender-based violence?
3. What are some of the causes behind sexual and gender-based violence in your community?
4. Who is most vulnerable to sexual and gender-based violence in your community?
5. What are some challenges that survivors of sexual and gender-based violence face?
    a. PROBE: physical, psychological, social
6. What are some coping strategies survivors of sexual and gender-based violence use?
7. What are community responses when sexual and gender-based violence occurs?
    a. What is done to help survivors?
8. What is done to prevent violence in your community?
    a. How could these efforts be improved?
    b. How could cultural teachings, stories or strengths be used to reduce violence?
9. What social and legal services exist to help survivors of violence?
    a. What are some barriers survivors might face when trying to access these services?
    b. What might be some ways to improve access or remove those barriers for survivors of sexual and gender-based violence? What kinds of things might make survivors more likely to look for or use services?
10. What would you like to see happen to reduce sexual and gender-based violence in your community?

Supplement to: Logie CH, Okumu M, Loutet M, et al. A participatory comic book workshop to improve youth-friendly post-rape care in a humanitarian context in Uganda: a case study. *Glob Health Sci Pract*. 2023;11(3). https://doi.org/10.9745/GHSP-D-22-00088

Interview Guide – Healthcare Providers

1. How long have you been working with youth in Bidi Bidi? What kind of services do you provide/what is your role?
2. What is the situation of sexual and gender-based violence like in Bidi Bidi settlement? Can you give examples of what sexual and gender-based violence might look like among people seeking health services?
3. What kind of healthcare provider training are you given regarding working with people who experienced sexual and gender-based violence? What about among youth aged 16-24?
4. Who is most vulnerable to sexual and gender-based violence from your perspective?
5. What are some challenges that survivors of sexual and gender-based violence face?
    a. PROBE: physical, psychological, social
6. What is the process like for receiving post-rape care in your clinic/setting? Specifically, please describe the process for accessing post-exposure prophylaxis.
    a. What are some barriers to survivors accessing this care? What are some facilitators?
    b. What might be some ways to improve access or remove those barriers for seeking healthcare among survivors of sexual and gender-based violence?
    c. What kinds of things might make survivors more likely to look for or use services?
    d. What is the screening process for sexual and gender based violence like at your clinic? (e.g. who is screened, what is the process after they are screened, what referrals are made)
7. What information and training would you like to help to provide healthcare to youth sexual and gender-based violence survivors?

Supplement to: Logie CH, Okumu M, Loutet M, et al. A participatory comic book workshop to improve youth-friendly post-rape care in a humanitarian context in Uganda: a case study. *Glob Health Sci Pract*. 2023;11(3). https://doi.org/10.9745/GHSP-D-22-00088

Interview Guide - Youth

1. What is the situation of sexual and gender-based violence like in Bidi Bidi settlement? Can you give examples of what sexual and gender-based violence might look like in your community? (inform participant they do not have to share their personal experiences of violence)
2. What about situations of sexual and gender-based violence in the journey to Uganda?
3. What are community attitudes toward sexual and gender-based violence?
4. What are some of the causes behind sexual and gender-based violence in your community?
5. Who is most vulnerable to sexual and gender-based violence in your community?
6. What are some challenges that survivors of sexual and gender-based violence face?
    a. PROBE: physical, psychological, social
7. What are some coping strategies survivors of sexual and gender-based violence use?
8. What are community responses when sexual and gender-based violence occurs?
    a. What is done to help survivors?
9. What is done to prevent violence in your community?
    a. How could these efforts be improved?
10. What social and legal services exist to help address these problems? Who provides these services?
11. How could these efforts be improved?
    a. What do you wish healthcare providers knew when they are giving services to violence survivors? How do you wish healthcare providers would treat violence survivors? What specific services do you think violence survivors could benefit from at a healthcare setting?
12. What would you like to see happen to prevention sexual and gender-based violence in your community? Can you think of any examples where you wish people knew how to prevent violence?
13. When someone experiences sexual and gender-based violence, how do you think people should support them? For instance, is there something they can say or do to be supportive? What might that look like for a friend, a brother, a sister, a parent, a community leader? (probe for different supportive reactions)

Supplement to: Logie CH, Okumu M, Loutet M, et al. A participatory comic book workshop to improve youth-friendly post-rape care in a humanitarian context in Uganda: a case study. *Glob Health Sci Pract*. 2023;11(3).
https://doi.org/10.9745/GHSP-D-22-00088

Supplement Table. Feedback from Health Care Providers Who Participated on Ngutulu Kagwero Workshops

Theme	Subtheme	Illustrative quotation
Workshop learning outcomes	Increased knowledge of sexual violence and supporting survivors	"I got additional knowledge on sexual and gender-based violence in the comic." (HCP, man)<br>"[I learned] "Guidelines and provision of PEP." (HCP, man)<br>"It has helped me a lot to be active, in order to identify the cases of gender-based violence. I gained knowledge on how to approach, how to manage cases of gender-based violence, especially how to handle the survivor. I've been very keen enough to try to screen for gender-based violence." (HCP, man)<br>"I learned a lot in SGBV management." (HCP, woman)<br>"Managing sexual and gender-based violence care, treat and refer to police and as medical interpreter I keep confidentiality of all cases. As an interpreter I am able to direct patients of sexual gender violence on how to get PEP and support." (HCP, man)<br>"How to help someone who has been raped by helping that person to go to the hospital fast so as to be tested for STDs." (HCP, man)<br>"I learned how to help a survivor come out of stigma." (HCP, woman)
Initiatives following workshops	Youth-centered strategies to promote privacy, SGBV training	"We created the youth friendly corners, special clinics and staff to attend to their needs." (HCP, man)<br>"Youth friendly corners in the hospital. Counseling sessions for youth twice a week." (HCP, woman)<br>"Youth friendly corner opportunity." (HCP, man)<br>"Giving youth a survivor space in a facility, whereby we offer SGBV services" (HCP, man)<br>"We have established youth friendly corner." (HCP, man)<br>"Carried out massive sensitization of youth friendly services in communities. Initiated life skills training for youths." (HCP, man)<br>"We're able to provide them privacy in any case we receive a client." (HCP, man)<br>"We are able to manage cases in a confidential manner." (HCP, woman)<br>"We provide health education to entire persons and our clients. Every morning, at least each and every morning, we have to, after completing the topic you're prepared, you have to echo something concerning sexual gender-based violence on daily basis." (HCP, woman)<br>"I gave the information to my colleagues, and we tried to display the rights so that the youth can understand their rights." (HCP, woman)<br>"We handled the youth and we were able to give them the services." (HCP, woman)
	HCP training	"More knowledge on PEP." (HCP, woman)<br>"I want to learn about sexual transmitted infections and sexual disease prevention and control." (HCP, man)

Supplement to: Logie CH, Okumu M, Loutet M, et al. A participatory comic book workshop to improve youth-friendly post-rape care in a humanitarian context in Uganda: a case study. *Glob Health Sci Pract*. 2023;11(3). https://doi.org/10.9745/GHSP-D-22-00088

Further support needed		"We need more trainings on SGBV and there is a need for vocational trainings." (HCP, woman)<br>"Guidelines to managing SGBV and maybe soft copies of comics for CMEs at facility." (HCP, man)
	Resources	"Cost facilitation in terms of money to support the implementation [of youth-friendly services]." (HCP, man)<br>"Books to give them, some medical supplies, then more comic books." (HCP, woman)<br>"Equipment for recreation activities." (HCP, man)<br>"Once issues of gender-based violence can be identified and brought to the hospital attention after discharge of the patient, the person should be declared, and the victim should be linked to other services like legal services, social worker for more counseling, so that they can get a comprehensive service." (HCP, man)<br>"There is a need for TV with entertainment. We also need stuff for youth. We need a separate laboratory for youth." (HCP, woman)<br>"We can also install a TV that will keep them around in the center there." (HCP, man)
	Further youth and community engagement	"We can make them form a drama group that can teach them about the sexual and gender-based violence." (HCP, man)<br>"I need to have more time with the youth and educate them about the ways of preventing the violence in our communities. Through dialogues and having meetings." (HCP, woman)<br>"We need some people or the health workers to give health education to the youth to encourage them about youth services and SGBV. We also encourage to reduce stigma through health education" (HCP, woman)<br>"SGBV prevention at the household level. Immediately this person, this plan can be sent to the counselor to probe for more information concerning the social issues at home that might be causing the gender-based violence." (HCP, man)<br>"Parent and youth interface programs to bridge the gap." (HCP, man)<br>"There is need to sensitize the community through dialogue, actually it is going on now but we need to sensitize them more." (HCP, woman)<br>"We still actually need to go out into the community to educate the community about SGBV and how to stop this." (HCP, man)<br>"Community awareness on the existence of the youth friendly services." (HCP, woman)<br>"I want to get out to the community; we need also the community or community members to be involved in the training session. These members will be able to pass that information to the community in the local languages, and let them know what it is, sexual gender-based violence related. At the end of the day people will understand." (HCP, man)
